# The efficacy of neuro-optometric visual rehabilitation therapy in patients with visual snow syndrome

**DOI:** 10.3389/fneur.2022.999336

**Published:** 2022-12-05

**Authors:** Terry Tsang, Charles Shidlofsky, Vanessa Mora

**Affiliations:** ^1^Dr. Terry Tsang Optometry, Inc., Irvine, CA, United States; ^2^Neuro-Vision Associates of North Texas, Plano, TX, United States; ^3^Department of Research, Visual Snow Initiative, Miami, FL, United States

**Keywords:** visual snow syndrome, visual snow, neuro-optometric rehabilitation, quality of life, visual function questionnaire

## Abstract

**Objective:**

This study intends to evaluate the feasibility of Neuro-Optometric Rehabilitation Therapy (NORT) to treat Visual Snow Syndrome (VSS). This pilot study utilized the National Eye Institute Visual Function Questionnaire (NEI-VFQ-25) to assess quality of life (QOL) before and after treatment.

**Methods:**

Twenty-one participants were recruited as successive intake patients diagnosed with VSS at the clinics of Dr. Terry Tsang Optometry, Inc and Neuro-Vision Associates of North Texas. Participants completed the NEI Visual Function Questionnaire 25-2000 edition and performed NORT, conducted by a neuro-optometrist or a qualified vision therapist. The NEI-VFQ-25 was administered before, at 6 weeks, and at 12 weeks of NORT to evaluate the effects of treatment on patient QOL.

**Results:**

The participants demonstrated an improvement in QOL composite and subscale scores after 6 and 12 weeks of NORT treatment. The NEI-VFQ-25 composite scores from the pre-test (M = 68, SD = 18) and at 6 weeks of treatment (M = 75, SD = 17) indicate an improvement in QOL [t (20) = 4.0, *p* = 0.0007]. The NEI-VFQ-25 composite scores from pretest to 12 weeks of treatment showed further improvements. This trend of improvement on NEI-VFQ-25 composite scores continued into the 12th week (M = 77, SD = 17) of treatment [t (20) = 4.5, *p* = 0.0002]. The subscales of general vision, distance activities, near activities, social functioning, mental health, role difficulties and dependency also showed improvement.

**Conclusion:**

Our results suggest that patients with VSS experience improvement in QOL in as little as 6 weeks, with further improvement by 12 weeks of NORT. This suggests NORT is an effective treatment option for managing the condition and improving QOL in patients with VSS, although a reduction in specific symptoms has yet to be demonstrated. This study provides justification that NORT warrants further investigation on VSS symptom reduction.

## Introduction

Visual Snow Syndrome (VSS) is a commonly misdiagnosed neurological condition characterized by mild to severe, persistent visual abnormalities (1). Its defining symptom is constant flashing dots or static throughout the visual field, known as Visual Snow (VS) (2). Diagnosis of VSS requires the existence of VS for more than 3 months, alongside at least two of the following symptoms: blue field entoptic phenomenon, floaters, nyctalopia, palinopsia, photopsia, and photophobia (3). A range of comorbidities are consistently reported in patients with VSS, including tinnitus, migraines with or without aura, depersonalization, anxiety, depression, concussion, and fibromyalgia (3). Irritability, brain fog, concentration difficulty, stress, paresthesia, and tremors are also frequently reported (4–6). VSS can occur in isolation or with the associated comorbidities (7). The pathophysiology of VSS is still unclear, but previous research has provided insight that the condition extends farther than a patient's clarity system, including alterations in different brain areas such as the temporal lobe, limbic system, and parietal lobe, which are associated with visual processing (8).

Many patients with VS have struggled with daily life because of the symptoms associated with the syndrome. Driving, reading, enjoying nature, and utilizing technology are some of the activities that patients report difficulties with (1). Going out at night, being in bright light areas, and exercising also affect them. According to most patients, their work, study, and social lives are affected (9). Consistent with previous research, in this study patients reported the same difficulties in their daily life. In this study, many VS patients also had reduced functional vision in the areas of oculomotor skills, accommodation, and binocular vision.

Evidently, patients with VSS report their Quality of Life (QOL) improves when symptom severity decreases (10). In this study, questions included in the NEI Visual Function Questionnaire 25-2000 edition (NEI-VFQ-25) represent a measurement of dimensions of self-reported vision health status in a specific focus group (11). This survey allowed the measurement on QOL in patients with VSS including domains such as, difficulty with near and distance vision activities, limitations in social functioning, role, dependency on others, mental health, driving difficulties, peripheral and color vision, and ocular pain (12).

This study hypothesizes that Neuro-Optometric Rehabilitation (NORT) improves QOL in patients with VSS using the NEI-VFQ-25. NORT is a set of neurosensory and neuromuscular exercises prescribed and supervised by a neuro-optometrist or physician to increase oculomotor function, speed, accuracy, integration to develop and enhance visual skills and processing (13, 14). This research provides vital information for all health care professionals who encounter VSS patients by providing an effective treatment option for managing the condition.

## Methods

We proposed that NORT improves QOL in patients with VSS. A NEI-VFQ-25 assessment of the effect of neuro-optometric rehabilitation on daily functioning was conducted prior to, 6 weeks after, and 12 weeks after therapy.

### Ethical approval

This study was conducted at Dr. Terry Tsang Optometry, Inc in Irvine, California, and Neuro-Vision Associates of North Texas in Plano, Texas. The study followed the requirements of the Western International Review Board, and each patient gave written informed consent compliant with the Health Insurance Portability and Accountability Act.

### Participants

There were 34 participants enrolled in the study between the two proposed locations, and 21 participants completed the study with the primary objective of obtaining preliminary results on whether using NORT for patients with VSS can improve QOL.

Participants with VSS were recruited from the Visual Snow Initiative (VSI) database. The control group participants were the same patient population before they received NORT treatment. All participants were previously diagnosed with VSS by a neurologist, ophthalmologist, neuro-optometrist, or neuro-ophthalmologist. Participants were also questioned about their medical history, medications, prescription glasses or contact lenses, and prior ophthalmological or imaging exams. Small participant recruitment and treatment discontinuation were due to the COVID-19 pandemic restrictions on travel, illness, and job loss. The exclusion participant criteria are lost follow-up, missed more than one treatment session, not able to come in for in-person sessions, and previously diagnosed with hallucinogen-persisting perceptual disorder (HPPD).

### Data analysis

The data analysis was conducted based on NEI-VFQ-25 data for 21 participants [mean (SD) age, 28 [12] years; range, 9–59 years; 52% male and 48% female]. Statistical Data were analyzed using GraphPad T test calculator by Dotmatics (15).

### Treatment protocol

All participants had in-office examinations utilizing standard optometric techniques to evaluate functional aspects of vision including, ocular motility, fixation, pursuits, and saccades, binocular vision, accommodation, and visual processing. Measuring tools included the Snellen eye chart, Wolff wands, +/-2.00 flippers, 3 BI/12 BO flippers, red lens, penlight, and prism bars. The Snellen chart measured all participants' monocular and binocular visual acuity. An objective measure of eye movements of the visual system was obtained using the electronic eye-tracking system. Participant binocular status, distance, and near phoria were assessed using distance or near fixation targets and prism bars. Divergence, convergence, and vertical ranges were acquired when the participant fixed at a distance and near target. The near point of convergence was measured using a red lens with a penlight and push up method. Accommodative status, both monocular and binocular, was assessed with the push-up method. Accommodative Facility, both monocular and binocular, was measured using +/- 2.00 Flippers. The Balance Tracking System measured sensory integration, proprioceptive input, visual input, and vestibular input on balance. During this evaluation, various lenses, prisms, and filters were used to determine if they were beneficial for therapeutic use during the therapy sessions. Cognitive testing, using either the MOCA or Cognivue System was administered. Before undergoing NORT, participants completed the NEI-VFQ-25.

Researchers and survey experts at the Research and Development (RAND) Corporation have developed numerous health-related surveys (11). A variety of public surveys are available that assess patient health and evaluate the quality of care and QOL (12). The surveys are typically designed for the general public but can also be for specific groups. The NEI-VFQ-25 was developed by RAND and funded by the National Eye Institute (NEI) to create a survey that would measure the dimensions of self-reported vision-targeted health status (11). The NEI-VFQ originally consisted of 51 items but was shortened to a 25-item questionnaire (NEI VFQ-25) which was shown to provide valid data when assessing vision-related QOL in several eye conditions (12). The NEI-VFQ-25 assesses the impact of visual impairment and visual symptoms on generic health categories, including emotional wellbeing, social functioning, and daily visual functioning (12). The NEI-VFQ-25 includes 25 items that measure the difficulty associated with specific visual symptoms and daily activities (12). Each item is assigned to one of the 12 subscales: general health, general vision, ocular pain, near activities, distance activities, social functioning, mental health, role difficulties, dependence, driving, color vision, and peripheral vision (16). The questionnaire was initially written in English and has been translated and adapted to many languages (11). The subscale and composite computations are scored on a scale of 0 to 100, with higher values indicating better outcomes of QOL (12). An overall composite score was calculated as the mean of all subscale scores and is assumed to be a unidimensional scale measuring QOL. Subscale scores were calculated as the mean of all component item scores.

**Figure 1:**
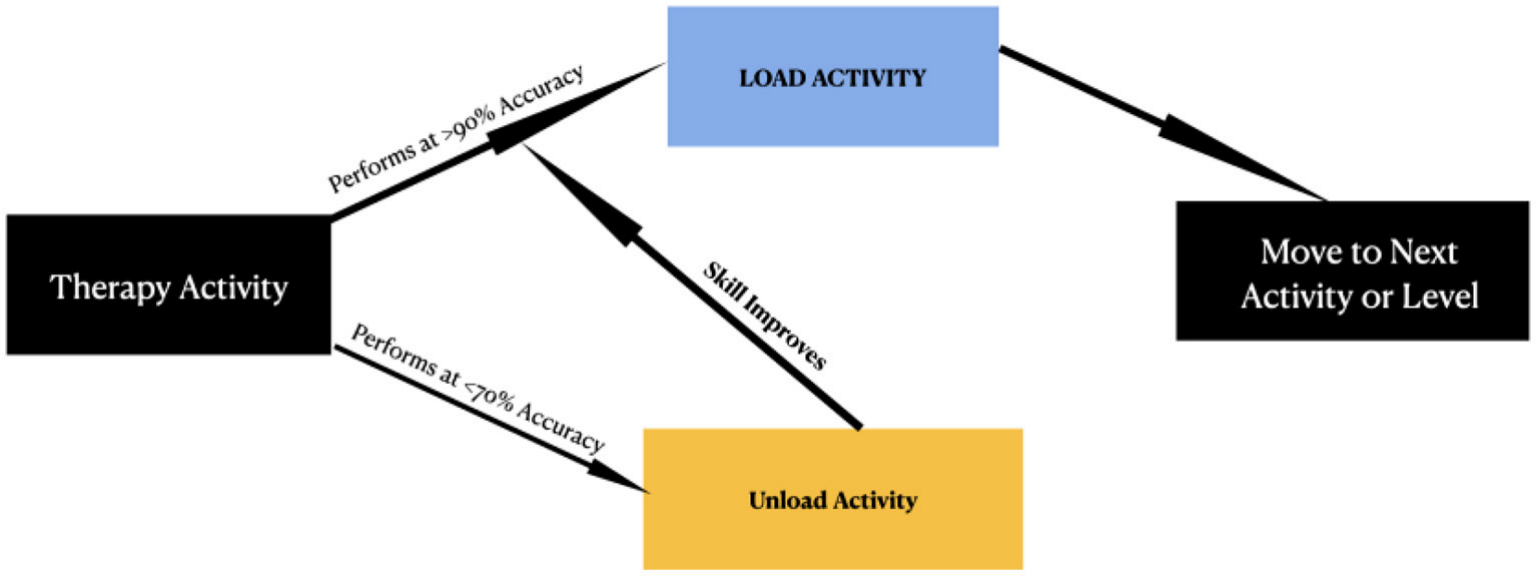
A decision tree for determining the next course of action for NORT activities.

NORT was implemented based on the individual's optometric findings such as accommodation, vergence, binocular vision skills and oculomotor skills. NORT therapeutic techniques were adapted from Applied Concepts in Vision Therapy (17). A summary of all basic categories and the list of activities within each category are reported in Table 1. For each activity utilized, a decision tree presented in Figure 1, was used for each patient to determine how to proceed to load, unload or move on to the next procedure or the next category. The different types of loading activities that were implemented were, adding balance, adding metronome, adding motion, and adding cognitive load/math.

**Table 1:** Summary of NORT activities used in this study with associated range of levels.

**Category**	**Description**	**Level 1**	**Level 2**	**Level 3**
**Motor skill, vestibular, peripheral awareness**				
Eye stretches	Develops proprioceptive awareness of the eyes in physical space			
McDonald chart	Develops peripheral awareness	Standing	Add balance	
Space fixator	Utilizes visual-spatial skills to help guide motor planning	Standard	Add beat	Add balance, yoked prism
4 Chart saccade	Develops high-level saccadic eye movements	Standard	Add beat	Add movement, and letter charts
2 chart wall Saccade with postural shift	Develops saccadic eye movements with weight shift and postural changes		Add- T pattern step	Add- Metronome beat
Bean bag toss- peripheral system	Bean bag catching using peripheral vision	Standard	Add beat	Add prisms 10 Prism diopters
Yoked prism walks–with prism glasses	Visually guided movements while wearing prism lenses	10 prism diopters	12 prism diopters	15 prism diopters
Light board–Binovi, Fit lights, Senaptec, SVI	These tools help to develop eye movements and visually guided motor movements			
Marsden ball	Improves spatial awareness which is the realization of the location, distance, and direction of objects in the environment related to each other and oneself			
Infinity walk	Develops and enhances the visual and vestibular connection			
Central peripheral saccades	Develops spatial awareness and accurate peripheral localization			
Optics trainer- visual spatial	Utilizes virtual reality hardware for Vision Therapy and Vision Training			
Photobiomodulation	Modulates the sympathetic and parasympathetic autonomic nervous system			
Balance activities on balance tracking systems (BTS)	Uses the visual system to guide shifts in the center of balance.			
**Central vision/ alignment**				
Monocular accommodative rock	Develops the ability of each eye to rapidly and accurately change visual focus at a near distance.			
Monocular hart chart rocks	Develops the focusing ability of the eyes			
Chalkboard	Ocular fixation centrally with integrated coordinated body movements.	Circles	Peripheral to central	
Bean bag battery	Improves visual-vestibular integration and eye-hand coordination while looking at the bean bag directly	Without prism	With prisms lenses	
Binocular accommodative rock	Develops the ability to rapidly and accurately change focus at a near distance using both eyes.	1.50	2.00	2.50
Brock string	Develops the binocular ability of the visual system in all gazes at varying distances			
Stereo activities vision therapy systems	Develops the binocular ability of the visual system at varying distances	VTS4 flat	VTS4 stereo	
**Vision skills**				
MFBF activities				
Touch Board. Binovi, Senaptec, fitlights, SVI	These computer-based therapeutic instruments are be used to enhance various visual abilities		Binovi ARC	R/B Saccades
Strobe glasses	Trains the connections between an individual's eyes, brain, and body	Bean bags	Tennis balls	Heco Stix
Optics trainer	Makes visual and sensory training immersive while providing effective treatment and metrics	General game activities	Multiple object tracking	
Gaze stabilization	To allow the eyes, inner ears, and brain to enhance processing	Single gaze	In multiple gaze fields	
3 Wall saccades	Improves the ability to organize and track visually while maintaining peripheral awareness			
Bean bag toss with 10 D yoked prism	The purpose of this activity is to enhance binocular sensory-motor awareness.			
**Visual perception skills**				
Set				
Q Bitz				
On the dot pattern				
Parquetry block				
Geoboard patterning				

NORT consisted of 12 one-on-one sessions, each being 60 min in duration. Each session included 3–5 exercises and visual home assignments. The home assignments were performed 5 days a week for 12 weeks and were monitored through follow-up visits. NORT was delivered either by a neuro-optometrist or trained vision therapist under the direction of a neuro-optometrist. Activities were customized for each patient to suit the participants' needs and followed the activities within the listed protocol. At 6 weeks, participants were reassessed using the NEI-VFQ-25, followed by a comprehensive reassessment at visit twelve, including optometric examination and the NEI-VFQ-25.

A brief description of each therapy technique is described as follows:

*Eye Stretches*: This activity aims to develop proprioceptive awareness of the eyes in physical space.

*McDonald Chart*: This activity aims to develop peripheral awareness.

*Space Fixator*: This activity aims to use visual-spatial skills to help guide motor planning.

*4 Chart Saccade*: This activity aims to perform high-level saccadic eye movements.

*Wall Saccade with Postural Shift- using a T Step*: This activity aims to dissociate focal visual processing from the spatial visual process enabling spatial movement and weight shift with visual fixations.

*Marsden Ball Batting*: This activity aims to improve spatial awareness which is the realization of the location, distance, and direction of objects in the environment related to each other and oneself.

*Infinity Walk*: This activity aims to develop and enhance the visual and vestibular connection.

*Central-Peripheral Saccades*: This activity aims to develop spatial awareness and accurate peripheral localization.

*Optics Trainer*: Optics Trainer VR utilizes virtual reality hardware for Vision Therapy and Vision Training. This system consists of 12 activities designed to make visual and sensory training fun and immersive while providing effective treatment and metrics.

*Biophoto Modulation*: We used specific tint wavelengths to modulate the sympathetic and parasympathetic autonomic nervous system. We used well-established Syntonic principles in choosing the appropriate tints for the individual patient.

*Balance Tracking Systems*: This activity aims to use the visual system to guide shifts in the center of balance.

*Chalkboard Circles*: This activity aims to develop a foundation of internal organization and control of body movement and develop the ability to integrate the top, bottom, and sides of the body in a coordinated manner.

*Bean Bag Battery*: The purpose is to improve visual-vestibular integration and eye-hand coordination.

*Monocular Accommodative Rock*: The purpose of this activity is to develop the ability of each eye to change visual focus rapidly and accurately at a near distance.

*Binocular Accommodative Rock*: This activity aims to develop the ability to change focus rapidly and accurately at a near distance using both eyes.

*Brock String*: The purpose of this exercise is to have both eyes pointing in the same place at the same time with no suppression of vision in either eye.

*Monocular in a Binocular Field activity*: This activity aims to develop accurate perception of details with one eye and the perception of the background with the other eye.

*Touch board: Binovi, Senaptec, Fitlights, Sanet Vision Integrator*: The Binovi, Senaptic, Fit light, and the Sanet Vision Integrator (SVI) are computer-based therapeutic instruments that can be used to enhance the following visual abilities: Pursuits, Saccades, Fixation stability, Eye-hand Coordination, Visual Reaction Time, Speed and Span of recognition, Automaticity, Contrast Sensitivity, plus Visual and Auditory Sequencing and Memory.

*Senaptec Strobes with Ball toss*: The Senaptec Strobes are designed to train the connections between an individual's eyes, brain, and body. The lenses use liquid crystal technology to flicker between transparent and opaque, removing visual information and allowing an individual to process more efficiently.

*Gaze Stabilization ExerciseVOR: Vestibular Ocular Reflex (VOR) Exercise*: These exercises allow the eyes, inner ears, and brain to enhance processing.

*Binocular Vision: Vision Therapy Systems*: Computer Orthoptics includes complex monocular and binocular stimuli, which allow automatic testing and measurement of the following skills: oculomotor (pursuits and saccades); fusional ranges; phorias; motor fields; fixation disparities, suppressions; retinal correspondence; accommodative facility; stereopsis, visual memory, and aniseikonia.

*Three Chart Wall Saccades*: This activity aims to improve the ability to organize and track visually while maintaining peripheral awareness.

*Bean Bag Tossing with yoked prism glasses*: The purpose of this activity is to enhance binocular sensory-motor awareness.

*Visual Perceptual Processing*: Visual processing is the ability to perceive, analyze, synthesize, and think with visual patterns. It involves the ability to store and recall information *via* visual imagery and visual memory. Using manipulatives such as those in the list below helps enhance visual processing skills.

a. Set.

b. Q Bitz.

c. On the dot.

d. Parquetry Block.

e. Geoboard patterning.

## Results

The composite and subscale scores of the NEI-VFQ-25 of all participants are listed in Table 2.

**Table 2:** Participants mean NEI-VFQ-25 composite and subscale scores before and after NORT treatment with associated effect size.

	**Before**	**6 Weeks**	***p*-Value**	**Effect size**	**12 Weeks**	***p*-Value**	**Effect size**
Composite score	68 ± 18	75 ± 17	0.00070	0.41	77 ± 17	0.00020	0.52
General health	59 ± 20	59 ± 14	0.97	NS	60 ± 18	0.86	NS
General vision	58 ± 17	65 ± 18	0.064	0.43	69 ± 15	0.0083	0.67
Ocular pain	74 ± 23	82 ± 18	0.11	NS	82 ± 18	0.056	NS
Near activities	73 ± 21	81 ± 15	0.010	0.43	83 ± 15	0.0016	0.57
Distance activities	68 ± 22	74 ± 20	0.0099	0.29	76 ± 22	0.0024	0.38
Social functioning	79 ± 21	84 ± 21	0.023	0.24	89 ± 15	0.00020	0.60
Mental health	38 ± 27	51 ± 28	0.00050	0.48	55 ± 30	0.00040	0.61
Role difficulties	52 ± 34	59 ± 33	0.037	0.22	64 ± 33	0.0028	0.34
Dependency	66 ± 35	74 ± 31	0.012	0.24	75 ± 31	0.011	0.29
Driving^**^	68 ± 14	73 ± 16	0.13	NS	80 ± 11	0.0056^**^	0.90
Color vision	98 ± 7	100 ± 0	0.16	NS	99 ± 5	0.33	NS
Peripheral vision	74 ± 29	80 ± 24	0.29	NS	77 ± 22	0.54	NS

** Driving was limited to 11 participants who were driving, NS, Not Significant.

The NEI-VFQ-25 composite scores from the pre-test (M = 68, SD = 18) and at 6 weeks of treatment (M = 75, SD = 17) indicate an improvement in quality of life [t (20) = 4.0, *p* = 0.0007]. This trend of improvement on NEI-VFQ-25 composite scores continued into the 12th week (M = 77, SD = 17) of treatment [t (20) = 4.5, *p* = 0.0002].

Increases in score for distance activities also occurred. The NEI-VFQ-25 distance activities scores from the pre-test (M = 68, SD = 22) and at 6 weeks of treatment (M = 74, SD = 20) indicate an improvement in quality of life [t (20) = 2.85, *p* = 0.0099]. This trend of improvement on NEI-VFQ-25 distance activities scores continued into the 12th week (M = 76, SD = 22) of treatment [t (20) = 3.47, *p* = 0.0024].

There was no significant increase in score for general vision at 6 weeks. The NEI-VFQ-25 general vision scores from the pre-test (M = 58, SD = 17) and at 6 weeks of treatment (M = 65, SD = 18) indicate no significant improvement in quality of life [t (20) = 1.96, *p* = 0.0636]. However, the NEI-VFQ-25 general vision scores from pretest to 12 weeks of treatment showed improvements, on NEI-VFQ-25 general vision scores continued into the 12th week (M = 69, SD = 15) of treatment [t (20) = 2.93, *p* = 0.0083].

The NEI-VFQ-25 near activities scores from the pre-test (M = 73, SD = 21) and at 6 weeks of treatment (M = 81, SD = 15) indicate an improvement in quality of life (t (20) = 2.83, p = 0.0104). This trend of improvement on NEI-VFQ-25 near activities score continued into the 12th week (M = 83, SD = 15) of treatment [t (20) = 3.64, *p* = 0.0016].

Table 2 also includes effect size calculations (Cohen's D) for every subscale where statistically significant improvement was shown after 6 and 12 weeks of NORT treatment. The data shows an upward trend for effect size in each case which indicates better QOL outcomes with 12 weeks vs. 6 weeks of NORT treatments.

Overall, the mean score of the participants QOL improved significantly after 6 and 12 weeks of treatment for composite, distance and near vision scores showing NORT has the potential to improve the QOL for patients suffering from VSS.

Additionally, a statistically significant improvement was observed at 12 weeks of NORT treatment compared to pre-6 weeks of treatment for social functioning, mental health, role difficulties and dependency. An improvement was also observed in the driving subscale after 12 weeks of NORT compared to pre-6 weeks of treatment, however the improvement was not statistically significant as driving was limited to only 11 participants.

No significant change was seen in general health, ocular pain, color vision, and peripheral vision. The results for general vision did not show improvement at 6 weeks, but showed improvement at 12 weeks, suggesting that it is possible that change is not immediate, and change over a longer time can occur.

## Discussion

The pathophysiology of VSS reaches beyond the visual system. This challenges physicians and researchers as conventional neuro- or ophthalmological tests appear normal. We proposed that NORT improves QOL in patients with VSS. We explored this proposal by applying the NEI-VFQ-25 before, at 6 weeks, and at 12 weeks of neuro-optometric rehabilitation to evaluate the effects of therapy and the impact on daily functions. This is the first study to assess the effect of NORT in treating VSS patient QOL.

Previous studies document VSS as having a negative impact on patient QOL and mental health (1). Treatment trials of anti-epileptic, migraine therapies, and acetazolamide on patients with VSS have shown limited to no efficacy with side effects often outweighing benefits (9). Consequently, people with VSS have difficulties with everyday activities including driving, reading, and using technological devices, causing problems with work, school, and socializing. Previous reports state the most common activity affected by VSS is driving, especially at night or in the dark. In addition, patients reported problems with reading, adding that they avoided it whenever possible (1). The use of screens was also a problem, preventing patients from working with technological devices (10). Other activities impacted included social activities, going out at night, physical activity, and enjoying natural scenery due to the temporary worsening of symptoms (1). According to our results, the post-treatment NEI-VFQ-25 composite scores, near and distance vision scores, social functioning, mental health, role difficulties, and dependency scores improved significantly following NORT. The reported debilitation of patients with VSS improved, and the activities could be performed effectively, suggesting an improvement in patient QOL.

In the areas of general health, ocular pain, color vision, and peripheral vision, there was no significant change as patients did not express any problems related to these functional elements at the outset of their diagnosis. Insignificant ocular pain, color vision, and patient's perception of their peripheral vision score changes might be due to the unremarkable impact of VSS in these areas.

Potential limitations of this study include recruitment bias, and a lack of objective measures of VSS severity. Patients who are more impacted by their symptoms may be more motivated to seek out and engage in clinical research, biasing studies toward reporting more severe cases. Additionally, some patients had to discontinue treatment due to COVID-19 pandemic restrictions on travel, sickness, and job loss.

Additionally, a symptom severity scale has been developed to measure the changes of VS parameters over time (18). However, a limitation of this study was that this measurable tool was not available at the time of the study design. In future, a NORT study involving both the NEI-VFQ-25 and the “Thirty-Day Static Diary” VS severity scale would be of interest.

## Conclusion

Using the NEI-VFQ-25 in participants with VSS, our results demonstrate an improvement in QOL post-6 and 12 weeks of NORT treatment. This suggests after NORT, patients could perform everyday activities effectively, improving work, study and social life. The results of this research provide vital information for all health care professionals who encounter patients with VSS by providing an effective treatment option for managing the condition and improving QOL. Our results suggest that patients with VSS experience improvement in QOL with NORT. This study provides justification that NORT warrants further investigation.

## Data availability statement

The original contributions presented in the study are included in the article/supplementary material, further inquiries can be directed to the corresponding author.

## Ethics statement

The studies involving human participants were reviewed and approved by Western International Review Board. Written informed consent to participate in this study was provided by the participants, or legal guardian/next of kin.

## Author contributions

TT and CS contributed to the conception and design of the study and handled the acquisition of data. TT and VM wrote the first draft of the manuscript. TT, CS, and VM supervised the project and were involved in critical revision of the manuscript. All authors contributed to the article and approved the submitted version.

## Funding

This study was fully funded by the Visual Snow Initiative.

## Conflict of interest

Author TT is employed by Dr. Terry Tsang Optometry, Inc. Author CS is employed by Neuro-Vision Associates of North Texas. The remaining author declares that the research was conducted in the absence of any commercial or financial relationships that could be construed as a potential conflict of interest.

## Publisher's note

All claims expressed in this article are solely those of the authors and do not necessarily represent those of their affiliated organizations, or those of the publisher, the editors and the reviewers. Any product that may be evaluated in this article, or claim that may be made by its manufacturer, is not guaranteed or endorsed by the publisher.
